# The Effect of Terlipressin on Renal Replacement Therapy in Patients with Hepatorenal Syndrome

**DOI:** 10.34067/KID.0000000000000132

**Published:** 2023-05-05

**Authors:** Juan Carlos Q. Velez, Florence Wong, K. Rajender Reddy, Arun J. Sanyal, Hugo E. Vargas, Michael P. Curry, Stevan A. Gonzalez, S. Chris Pappas, Khurram Jamil

**Affiliations:** 1 Department of Nephrology, Ochsner Health, New Orleans, Louisiana; 2 Ochsner Clinical School, The University of Queensland, Brisbane, Queensland, Australia; 3 Department of Medicine, University of Toronto, Toronto, Ontario, Canada; 4 Division of Gastroenterology and Hepatology, University of Pennsylvania Perelman School of Medicine, Philadelphia, Pennsylvania; 5 Division of Gastroenterology, Hepatology and Nutrition, Department of Internal Medicine, Virginia Commonwealth University, Richmond, Virginia; 6 Division of Gastroenterology and Hepatology, Mayo Clinic, Phoenix, Arizona; 7 Department of Medicine, Beth Israel Deaconess Medical Center, Boston, Massachusetts; 8 Department of Medicine, Baylor Scott & White All Saints Medical Center, Fort Worth, Texas; 9 Orphan Therapeutics LLC, Longboat Key, Florida; 10 Mallinckrodt Pharmaceuticals, Bridgewater, New Jersey

**Keywords:** AKI and ICU nephrology, AKI, cirrhosis, dialysis, ESLD, hemodialysis, HRS-AKI, RRT, renal failure, vasoconstrictor

## Abstract

**Key Points:**

- Hepatorenal syndrome type 1 (HRS-1) is an often fatal, but potentially reversible, kidney failure in patients with decompensated cirrhosis.
- Treatment with terlipressin in patients with HRS-1 is associated with a reduction in the need for RRT.

**Background:**

Hepatorenal syndrome type 1 (HRS-1)—also known as hepatorenal syndrome-AKI (HRS-AKI)—is a rapidly progressing and usually fatal, but potentially reversible, kidney failure occurring in patients with decompensated cirrhosis. A large proportion of patients with HRS-1 require renal replacement therapy (RRT). Terlipressin demonstrated efficacy in reversing HRS and improving renal function in patients with HRS-1 in three phase III, randomized, clinical trials (RCTs; *i.e.*, OT-0401, REVERSE, and CONFIRM). However, these RCTs were not designed to evaluate the effect of terlipressin on the requirement of RRT. In this study, the effect of terlipressin on RRT requirements in the pooled phase III patient population was assessed.

**Methods:**

For this retrospective analysis, data from patients who participated in the OT-0401, REVERSE, and CONFIRM studies were integrated in the largest-to-date randomized database (*N*=608).

**Results:**

The need for RRT was significantly decreased in patients in the terlipressin group versus the placebo group by day 30 (28.1% versus 35.9%, respectively; *P* = 0.040) and day 60 (30.1% versus 37.9%, respectively; *P* = 0.045) in the pooled population and also postliver transplantation (LT) at day 60 (20.5% versus 40.3%, respectively; *P* = 0.008) and day 90 (25.3% versus 43.1%, respectively; *P* = 0.018). More patients were alive and RRT-free by day 90 in the overall population (36.9% versus 28.5%; *P* = 0.030) and among patients who received an LT (60.0% versus 39.7%; *P* = 0.010). Random assignment to receive terlipressin was an independent positive predictor of avoidance of RRT (*P* = 0.042); while higher baseline serum creatinine (sCr) level and Child-Pugh scores were negatively associated with RRT avoidance (*P* < 0.001 and *P* = 0.040, respectively).

**Conclusions:**

Terlipressin decreased the requirement of RRT compared with placebo among patients with HRS-1, including those receiving LT. A lower sCr level at the beginning of therapy was associated with avoidance of RRT.

## Graphical abstract

**Figure:**
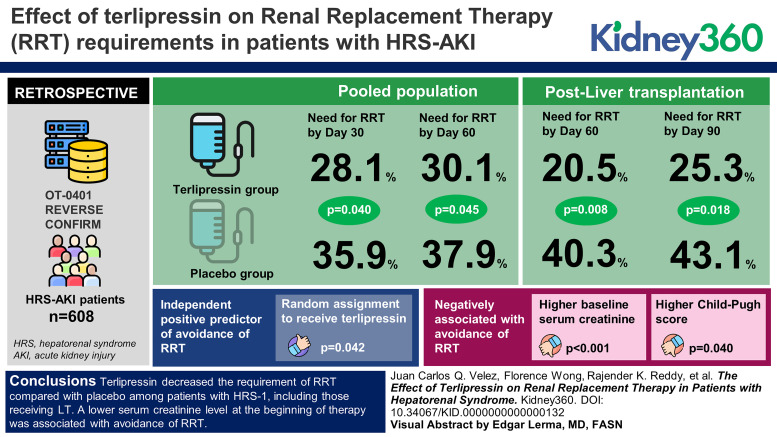

## Introduction

Hepatorenal syndrome type 1 (HRS-1)—also known as hepatorenal syndrome-AKI (HRS-AKI)—is a rapidly progressing and often fatal, but potentially reversible, deterioration in renal function that is due, in part, to a functional hemodynamic reduction in renal arterial perfusion seen in patients with decompensated cirrhosis.^1–3^

Patients with HRS-1 have marked morbidity and a very poor survival (*i.e.*, weeks to months) if left untreated.^4,5^ Liver transplantation (LT) is the only definitive treatment for HRS-1 because it eliminates the pathophysiological environment causing intrarenal vasoconstriction that leads to HRS-1.^1,6^ Pharmacological treatment with vasoconstrictor therapy may improve kidney function, reverse hepatorenal syndrome (HRS), and extend short-term survival, which is important for patients who are on the LT waiting list.^1^ Renal failure requiring renal replacement therapy (RRT) in patients with HRS-1, including those who are LT candidates, is a serious complication and portends an especially poor prognosis, with a 6-month mortality rate of over 80% in the absence of an LT.^2,5^ Pharmacological intervention to reduce the need for RRT in these patients is of significant clinical importance.

Terlipressin, a synthetic long-acting vasopressin analog, exerts a rapid vasopressor effect leading to splanchnic vasoconstriction and, consequently, an increase in effective intravascular volume and improved kidney function.^7,8^ Guidelines from both the European Association for the Study of the Liver and the American Association for the Study of Liver Diseases recommend vasoconstriction therapy with terlipressin plus albumin to treat patients with HRS-1.^2,9^ Furthermore, terlipressin is the only pharmacological agent approved by the US Food and Drug Administration for the treatment of adult patients with HRS.^10^

In three randomized, placebo-controlled, phase III studies (OT-0401 [ClinicalTrials.gov identifier: NCT00089570], REVERSE [ClinicalTrials.gov identifier: NCT01143246], and CONFIRM [ClinicalTrials.gov identifier: NCT02770716]), terlipressin was associated with higher rates of HRS reversal—defined as at least 1 serum creatinine (sCr) measurement of ≤1.5 mg/dl while on treatment—and improved renal function versus placebo.^11–13^ The need for RRT is an additional important and clinically meaningful end point that has not been previously explored in detail. The effect of terlipressin on RRT requirements in patients undergoing LT is of particular interest because peri-LT RRT is associated with poor outcomes post-transplant.^14^ Using data from the largest-to-date, pooled patient population from the three phase III studies (*i.e.*, OT-0401,^11^ REVERSE,^12^ and CONFIRM^13^) facilitated evaluation of the effect of terlipressin therapy on RRT and assessment of baseline predictors of avoidance of RRT.

## Methods

**Figure 1.**
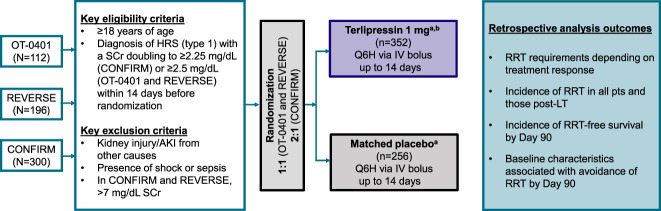
**Study design.** ^a^Concomitant albumin was strongly recommended at a dose of 100 g on day 1 and then 25 g/d until EOT in OT-0401^11^; 20–40 g/d in REVERSE^12^; and 1 g/kg to a maximum of 100 g on day 1 and 20–40 g/d thereafter in CONFIRM.^13^ ^b^If, after day 3, sCr had decreased, but by <30%, then the terlipressin dose could be increased to 2 mg Q6H. HRS, hepatorenal syndrome; sCr, serum creatinine; Q6H, every 6 hours; IV, intravenous; RRT, renal replacement therapy; pts, patients; LT, liver transplantation; EOT, end of treatment.

Data from patients who participated in the OT-0401,^11^ REVERSE,^12^ and CONFIRM^13^ studies were pooled for this *post hoc* analysis. The study design and methods for each trial were previously reported.^11–13^ In brief, eligible patients were adults with HRS-1 who were diagnosed using standard criteria before the adoption of the 2015 International Club of Ascites HRS-AKI criteria.^15^ Terlipressin (1 mg every 6 hours) or placebo was administered through intravenous bolus injection for up to 14 days. If, after day 3, sCr levels had decreased—but by <30%—then the terlipressin dose could be increased to 2 mg every 6 hours. Concomitant albumin was strongly recommended (CONFIRM: 1 g/kg body weight to ≤100 g, followed by 20–40 g/d; REVERSE: 20–40 g/d; OT-0401: 100 g on day 1 followed by 25 g/d). Figure 1 summarizes the study design of the three phase III trials.

RRT was defined as any procedure to replace nonendocrine kidney function (*i.e.*, intermittent hemodialysis and continuous RRT, including continuous hemofiltration, continuous hemodialysis, continuous hemodiafiltration, peritoneal dialysis, and other dialysis techniques). Outcomes were evaluated in the intent-to-treat (ITT) population, and in those who received an LT, up to 90 days from the first treatment dose. Patients who received simultaneous kidney and liver transplants were excluded from the post-LT outcomes analysis.

The need for RRT was evaluated with respect to response to terlipressin therapy. Complete response (CR) was defined as HRS reversal (*i.e.*, an sCr level of ≤1.5 mg/dl while receiving terlipressin or placebo by day 14 or discharge); partial response (PR)—defined here as a >30% reduction in sCr but not achieving HRS reversal; and no response (NR)—defined as a ≤30% reduction in sCr from baseline to the end of treatment.

### Statistical Analyses

To compare binary and categorical data, a chi-squared test was used if the number of events per cell was at least five; a Fisher exact test was used if the number of events per cell was <5. Logistic regression analysis was used to evaluate associations between baseline characteristics and RRT avoidance by day 90. First, baseline characteristics were analyzed in a univariable model for significant associations; then, all significant variables from the univariable analysis were included in a multivariable analysis with stepwise selection to determine a final explanatory model. All statistical tests were two-sided with a final significance level of 0.05. Statistical analyses were performed using Statistical Analysis Software version 9.4 (Cary, NC).

## Results

### Baseline Characteristics

In the pooled ITT population (*N*=608), 352 and 256 patients were randomly assigned to receive terlipressin and placebo, respectively. Baseline patient characteristics were similar between treatment groups and reflected the presence of advanced liver disease (Table 1; Supplemental Table 1): Mean (SD) Model for End-stage Liver Disease (MELD) scores were 33.0 (SD 6.4) and 33.1 (SD 5.9) in the terlipressin and placebo groups, respectively; over 65% of the patients in both groups had Child-Pugh-Turcotte class C (scores between 10−15). Acute-on-chronic liver failure grade 2−3 occurred in 53.4% of patients in the terlipressin group and in 55.1% of patients in the placebo group (Table 1).

**Table 1.** Baseline patient demographics and clinical characteristics; pooled intent-to-treat population^a^

Baseline Characteristics	Terlipressin (*n*=352)	Placebo (*n*=256)
**Age, yr, mean (SD)**	54.0 (10.6)	54.0 (10.5)
**Sex, male**	213 (60.5)	165 (64.5)
**Race**		
American Indian or Alaskan Native	3 (0.9)	4 (1.6)
Asian	8 (2.3)	1 (0.4)
Black	24 (6.8)	14 (5.5)
Native Hawaiian or Other Pacific Islander	0	1 (0.4)
White	313 (88.9)	235 (91.8)
**Alcoholic hepatitis present**	121 (34.4)	84 (32.8)
**sCr**		
Mean (SD), mg/dL	3.6 (1.3)	3.7 (1.1)
<3 mg/dl	126 (35.8)	84 (32.8)
≥3 to <5 mg/dl	182 (51.7)	139 (54.3)
≥5 mg/dl	44 (12.5)	33 (12.9)
**MELD score, *n***	312	221
Mean (SD)	33.0 (6.4)	33.1 (5.9)
**ACLF, *n***	352	255
Grade 0–1	164 (46.6)	114 (44.5)
Grade 2	116 (33.0)	92 (35.9)
Grade 3	72 (20.5)	49 (19.1)
**Child-Pugh-Turcotte class, *n***	337	242
Class A (5−6)	5 (1.4)	3 (1.2)
Class B (7−9)	100 (28.4)	71 (27.7)
Class C (10−15)	232 (65.9)	168 (65.6)
**MAP, *n***	352	255
Mean (SD), mm Hg	77.3 (12.0)	76.6 (10.9)
**Bilirubin, *n***	338	249
Mean (SD), mg/dL	12.8 (12.7)	14.1 (14.6)
**SIRS subgroup^b^**	112 (37.8)	78 (39.0)
**Etiology of cirrhosis**		
Alcohol	212 (60.2)	150 (58.6)
Hepatitis B	11 (3.1)	5 (2.0)
Hepatitis C	90 (25.6)	68 (26.6)
Primary biliary cirrhosis	11 (3.1)	7 (2.7)
Autoimmune hepatitis	13 (3.7)	9 (3.5)
Nonalcoholic steatohepatitis	52 (14.8)	36 (14.1)
**Hepatocellular carcinoma, present**	24 (6.8)	24 (9.4)
**Esophageal varices, present**	187 (53.1)	141 (55.1)
**Ascites, present**	347 (98.6)	247 (96.5)

Data are presented as *n* (%), unless otherwise noted. sCr, serum creatinine; MELD, Model for End-stage Liver Disease; ACLF, acute-on-chronic liver failure; MAP, mean arterial pressure; SIRS, systemic inflammatory response syndrome.

a The pooled population is from OT-0401,^11^ REVERSE,^12^ and CONFIRM.^13^

b SIRS subgroup data were available for the REVERSE^12^ and CONFIRM^13^ studies only.

### RRT and Treatment Response

A CR (*i.e.*, HRS reversal) was observed in 117/352 (33.2%) patients in the terlipressin group and in 42/256 (16.4%) patients in the placebo group (*P* < 0.001). In the combined treatment population, the need for RRT up to day 90 was significantly reduced in patients who experienced a CR (*n*=159) compared with NR ([*n*=392]; *P* < 0.001 at days 30, 60, and 90; Table 2). A significantly lower incidence of RRT was also observed in patients with a PR (*n*=75) versus NR (*P* < 0.001 at day 30; *P* = 0.012 at day 60; and *P* = 0.018 at day 90; Table 2). Furthermore, there were patients who achieved a CR both among those who needed RRT and those who did not need RRT; the median time to a CR was 6 days in both groups, ranging from 1 to 15 days in the no-RRT subgroup and 3–14 days in the RRT subgroup, respectively. However, as expected, there were significantly more patients who had a CR among those who did not need RRT: By day 15, 141/392 (36.0%) patients in the no-RRT subgroup achieved HRS reversal versus 18/206 (8.7%) patients in the RRT subgroup (*P* = 0.001).

**Table 2.** RRT requirement by treatment response; overall pooled intent-to-treat population^a^

Incidence of RRT by	CR (*n*=159)	PR (*n*=57)	NR (*n*=392)	*P* Value^b^	*P* Value^c^
Day 30	14 (8.8)	10 (17.5)	167 (42.6)	<0.001	<0.001
Day 60	16 (10.1)	15 (26.3)	172 (43.9)	<0.001	<0.012
Day 90	18 (11.3)	16 (28.1)	175 (44.6)	<0.001	<0.018

Data are presented as *n* (%). Complete response (*i.e.*, hepatorenal syndrome reversal) was defined as any serum creatinine level ≤1.5 mg/dl or less while receiving terlipressin or placebo. Partial response was defined as a >30% reduction in serum creatinine from baseline to the end of treatment; no response was defined as a ≤ 30 % reduction in serum creatinine from baseline to the end of treatment; hepatorenal syndrome reversal and partial response are mutually exclusive. RRT, renal replacement therapy; CR, complete response; PR, partial response; NR, no response.

a Population pooled from OT-0401,^11^ REVERSE,^12^ and CONFIRM^13^ studies.

b *P* value comparing complete response with no response.

c *P* value comparing partial response with no response.

### Incidence of RRT

**Figure 2.**
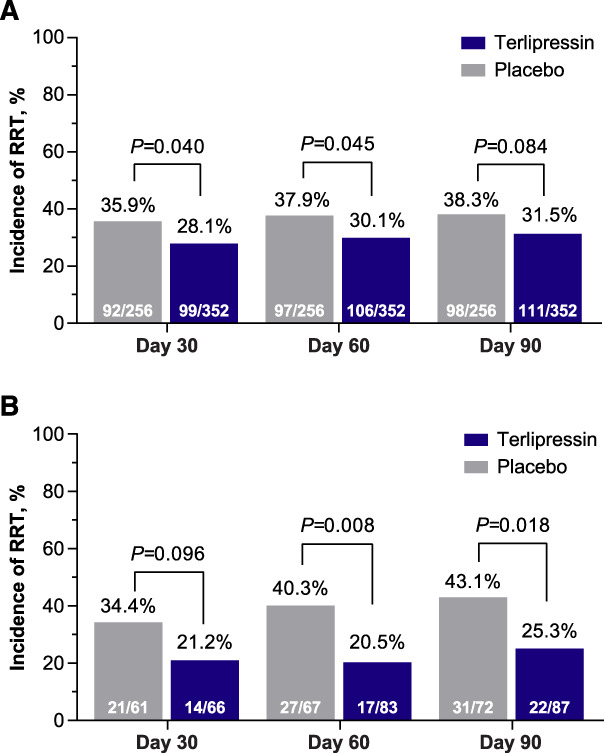
**Incidence of RRT decreases in the terlipressin vs placebo group.** (A) all patients and (B) RRT post-LT^a,b^; pooled ITT population^c^. ^a^Patients with a simultaneous liver-kidney transplant were excluded. ^b^For percentages, the denominator is the number of patients who had an LT, and the numerator is the number of patients who had RRT after the LT, calculated from day 1 to each time point. ^c^Population pooled from OT-0401,^11^ REVERSE,^12^ and CONFIRM^13^ studies. ITT, intent-to-treat.

Overall, the need for RRT was significantly decreased in patients in the terlipressin group versus the placebo group by day 30 (28.1% versus 35.9%, respectively; *P* = 0.040) and day 60 (30.1% versus 37.9%, respectively; *P* = 0.045); by day 90, the incidence of RRT was 31.5% in the terlipressin group versus 38.3% in the placebo group (*P* = 0.084; Figure 2A).

Importantly, post-LT, the incidence of RRT was significantly smaller in the terlipressin group versus the placebo group at days 60 and 90 after the first study drug dose (20.5% versus 40.3%, *P* = 0.008 and 25.3% versus 43.1%, *P* = 0.018, respectively; Figure 2B).

### Incidence of RRT-Free Survival

**Figure 3.**
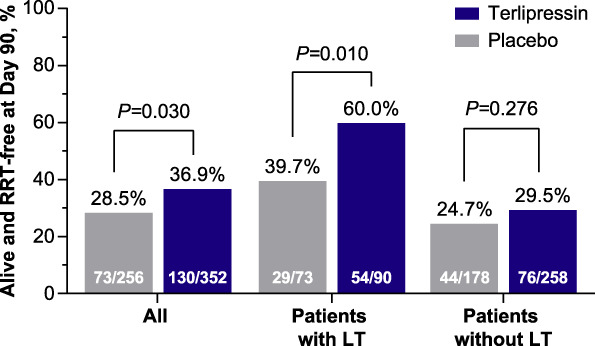
**Incidence of RRT-free survival at day 90; pooled ITT population.**^**a,b**^ ^a^When evaluating patients with an LT, those who had a simultaneous liver kidney transplant were excluded. ^b^Population pooled from OT-0401,^11^ REVERSE,^12^ and CONFIRM^13^ studies.

More patients who were randomly assigned to the terlipressin group (versus placebo) were alive and RRT-free by day 90 in the entire pooled ITT population (36.9% versus 28.5%; *P* = 0.030) and among those patients who received an LT (60.0% versus 39.7%; *P* = 0.010); Figure 3. However, there was no significant difference in the incidence of RRT-free survival among patients who did not receive an LT (29.5% and 24.7% in the terlipressin and placebo groups, respectively; *P* = 0.276); Figure 3.

### Liver Transplant Rate and Mortality

**Figure 4.**
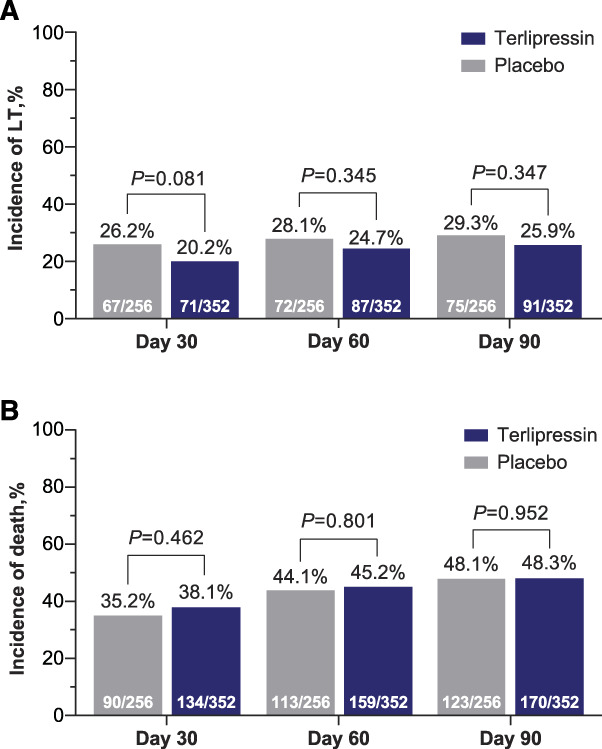
**LT and death incidences were similar in the terlipressin and placebo groups; pooled ITT population.^a^** (A) LT and (B) death. ^a^Population pooled from the OT-0401,^11^ REVERSE,^12^ and CONFIRM^13^ studies.

A similar proportion of patients received an LT in both treatment groups at each time point (day 30: 20.2% versus 26.2%, *P* = 0.081; day 60: 24.7% versus 28.1%, *P* = 0.345; and day 90: 25.9% versus 29.3%, *P* = 0.347, in the terlipressin and placebo groups, respectively; Figure 4A).

The mortality rate during the observation period was also comparable between the two treatment groups (day 30: 38.1% versus 35.2%; *P* = 0.462; day 60: 45.2% versus 44.1%, *P* = 0.801; and day 90: 48.3% versus 48.1%, *P* = 0.952, in the terlipressin and placebo groups, respectively; Figure 4B).

### Predictors of Avoidance of RRT

Predictors of RRT avoidance were evaluated in the pooled ITT population but excluded those patients who died before receiving RRT to avoid the competing effect of death on the resultant data; 412 patients (terlipressin, *n*=241; placebo, *n*=171) were included in this analysis.

**Figure 5.**
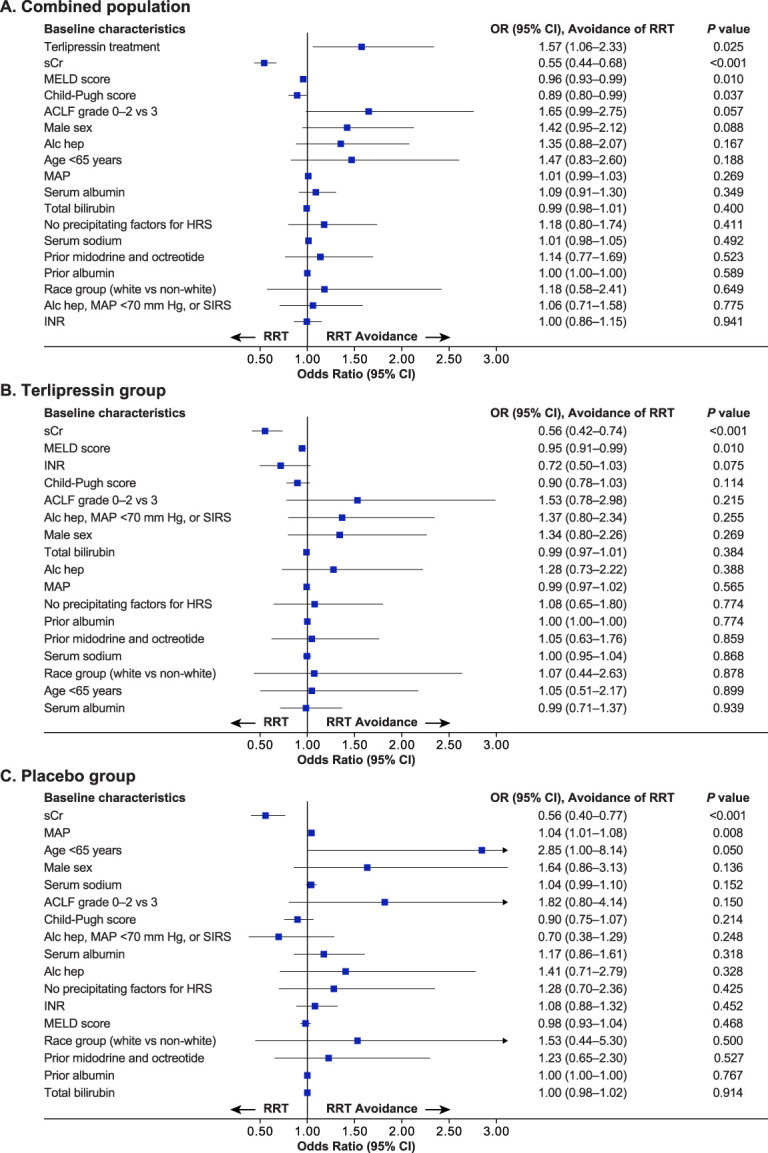
**Univariable analysis of the association between baseline characteristics and RRT avoidance by day 90, pooled ITT population, excluding patients who died without receiving RRT** in (A) Combined treatment groups; (B) terlipressin group; and (C) placebo group. OR, odds ratio; CI, confidence interval; ACLF, acute-on-chronic liver failure; Alc Hep, alcoholic hepatitis; MAP, mean arterial pressure; INR, international normalized ratio; SIRS, systemic inflammatory response syndrome; MELD, Model for End-Stage Liver Disease.

In the combined treatment population, random assignment to the terlipressin group was associated with a significantly greater odds of RRT avoidance (odds ratio [OR], 1.57; *P* = 0.025) (Figure 5A). Furthermore, higher sCr levels at study entry (OR, 0.55; *P* < 0.001) and higher scores for liver disease severity (MELD score: OR, 0.96, *P* = 0.010 and Child-Pugh score: OR, 0.89, *P* = 0.037) were negative predictors of RRT avoidance in the combined treatment group (Figure 5A).

In both treatment groups, a higher baseline sCr level was a negative predictor of RRT avoidance (OR, 0.56; *P* < 0.001, in both cases; Figure 5, B and C). In addition, in the terlipressin group, a higher MELD score was associated with an increased need for RRT (OR, 0.95; *P* = 0.010; Figure 5B), while in the placebo group, a higher baseline mean arterial pressure (MAP) was associated with avoidance of RRT (OR, 1.04; *P* = 0.008; Figure 5C).

Significant variables from the univariable analysis (*i.e.*, random assignment to terlipressin treatment, baseline sCr level, and Child-Pugh score) were included in the multivariable analysis with stepwise selection. Of note, the sCr value is included as a component in the MELD score calculation, along with bilirubin and international normalized ratio^16^; however, the latter two variables were not significant in the univariable analysis. Therefore, only baseline sCr values (and not MELD score, to avoid collinearity) were included in the multivariable analysis (as a continuous variable). The results confirmed that assignment to the terlipressin group was independently associated with avoidance of RRT (*P* = 0.042), while higher baseline sCr levels and Child-Pugh scores were associated with the need for RRT (*P* < 0.001 and *P* = 0.040, respectively; Table 3).

**Table 3.** Multivariable analysis of baseline characteristics associated with RRT avoidance by day 90; pooled intent-to-treat population,^a^ excluding those patients who died without receiving RRT

Baseline Parameter^a^	Combined Treatment Group (*n*=412)		
	*n*	OR (95% CI)	*P* Value
Terlipressin treatment group	412	1.56 (1.02 to 2.39)	0.042
sCr	412	0.52 (0.42 to 0.66)	<0.001
Child-Pugh score	393	0.89 (0.80 to 0.99)	0.040

OR, odds ratio; CI, confidence interval; sCr, serum creatinine.

a Population pooled from OT-0401,^11^ REVERSE,^12^ and CONFIRM^13^ studies.

b Because serum creatinine is a component of the Model for End-stage Liver Disease score, despite being significant in the univariable analysis, the Model for End-stage Liver Disease score was not included in the multivariable analysis.

## Discussion

Patients who were randomly assigned to terlipressin in the OT-0401, REVERSE, and CONFIRM phase III studies had a statistically significant improvement compared with placebo in kidney function, defined as a decrease in sCr or by achieving HRS reversal (an end point based on a numerical reduction in sCr concentration to a predefined level of <1.5 mg/dl).^11–13^ Although this end point is clinically important, it does not address the most serious short-term consequence of kidney failure in the context of any AKI—an acute need for RRT. Unlike vasopressor therapy, RRT in the setting of HRS-1—while temporarily improving acidosis, electrolyte imbalance, fluid overload, and/or uremia—does not address the fundamental pathogenesis of HRS-1, does not reverse this condition, and is typically reserved for patients who are considered as LT candidates or as a time-limited therapeutic trial.^2,17–19^ At the same time, RRT is an invasive and expensive intervention with a marked incidence of adverse events.^20^ Risks associated with RRT in patients with HRS-1 include typical acute complications related to venous access, hypotension during dialysis, and an increased risk of cardiac events.^5,21^ Moreover, patients with HRS-1 and cirrhosis have additional inherent challenges during RRT, which are related to the underlying pathophysiology of HRS-1 (*e.g.*, portal hypertension, decreased effective arterial blood volume, and decreased systemic vascular resistance with low MAP).^5^ Consequently, patients with HRS-1 commonly require admission to the intensive care unit and administration of continuous RRT.^5^ Peri-LT RRT is a negative prognostic factor for LT outcomes. For example, in a retrospective cohort study of patients with decompensated cirrhosis and HRS-1 (*N*=62), the duration of pretransplant dialysis was the only predictor of HRS nonreversal, with each additional day of pretransplant dialysis corresponding to a 6% increase in the risk of nonreversal.^22^ RRT post-LT in patients with HRS-1 is a profound risk factor for chronic RRT dependency and death in LT recipients. A large retrospective study (*N*=743) showed that post-transplant dialysis within 90 days after an LT was the strongest risk factor for progression to the need for chronic dialysis (relative risk=30.5), and only 37% of patients who needed RRT post-LT survived for 5 years after the first transplant.^23^ Conversely, patients who achieved HRS reversal with vasopressor therapy alone (*i.e.*, without RRT) experienced good post-transplant outcomes, similar to those patients without HRS-1 who received a transplant.^24^ Collectively, surviving without the need for RRT is associated with reduced morbidity, better quality of life, and improved outcomes in patients with HRS-1, including LT recipients.

The results presented here align with the previously published data regarding the effectiveness of terlipressin to improve kidney function and reverse HRS in patients with HRS-1.^11–13^ Moreover, this study provides new evidence indicating that terlipressin treatment, compared with placebo, reduces RRT requirements overall and, importantly, post-LT (Figure 2). The significant effect of terlipressin on the avoidance of RRT is further supported by the multivariable analysis: Randomization to the terlipressin group was an independent predictor of avoiding RRT by day 90. Furthermore, the incidence of RRT-free survival was higher in the terlipressin group overall and among patients who received an LT; however, in the absence of an LT, the number of patients alive without RRT by day 90 was similar (Figure 3), highlighting the importance of an LT as the only definitive long-term treatment for patients with HRS-1. While terlipressin can improve outcomes for renal function and, potentially, improve short-term survival, it does not cure the underlying liver disease. A similar proportion of patients in both treatment groups received an LT (Figure 4A). Therefore, it is not surprising that mortality was also similar among patients who were randomly assigned to receive terlipressin or placebo (Figure 4B).

Among those who achieved a CR (HRS reversal), the median time to a CR (*i.e.*, 6 days) was not different among those who ultimately avoided RRT compared with those who needed RRT at a later time point during the study period. As expected, there were significantly more patients who achieved a CR among those who did not need RRT compared with those who did need it (*P* < 0.001). These results support the data presented in Table 2, showing a negative correlation between treatment response and the need for RRT. While a PR—defined as a >30% reduction in sCr but not reaching a CR—may be perceived as a softer clinical end point compared with a reduction in the requirement for RRT, it is important to note that achievement of a PR or a CR was significantly associated with avoidance of RRT, thereby validating the clinical relevance of those prespecified outcomes. A >30% reduction in sCr may be a valuable end point that can be used in future clinical trials in patients with HRS-1.

Baseline factors associated with improved kidney function (defined by a reduction in sCr level) were previously evaluated. A lower baseline MELD score and lower sCr level were associated with HRS reversal in the OT-0401 and REVERSE studies; in addition, in REVERSE, baseline serum bilirubin was also identified as a significant factor associated with improved kidney function.^11,12^ In this analysis, we directly evaluated predictors of avoidance of RRT. In the univariable analyses, baseline characteristics of a more advanced stage of liver disease (*i.e.*, higher sCr level, MELD score, and baseline Child-Pugh score) were associated with a greater need for RRT by day 90 in the overall population. As expected, a higher baseline sCr was also a negative predictor of RRT avoidance in both the terlipressin and placebo groups, when treatment groups were analyzed separately. In addition, a higher MELD score was a negative predictor of RRT avoidance in the terlipressin group, while higher baseline MAP—likely indicating a less-severe hemodynamic derangement from HRS-1—was positively associated with RRT avoidance in the placebo group.

The multivariable analysis confirmed the significance of baseline sCr level and Child-Pugh score as independent factors associated with the need for RRT. These findings were not unexpected, as more advanced liver disease—characterized by a higher Child-Pugh score and higher sCr level—is associated with a more severe AKI and, consequently, a greater risk of the need for RRT.^2,25^ As recently shown by Curry *et al.*,^26^ more patients with a sCr level of <5 mg/dl stay alive and RRT-free through day 30 compared with the sCr ≥5 mg/dl subgroup (<3 mg/dl, 77.3%; ≥3 to <5 mg/dl, 76.5%; ≥5 mg/dl, 40.0%; *P* = 0.01). Therefore, early initiation of treatment with terlipressin, while sCr levels are relatively low, may reduce the need for RRT in patients with AKI because of HRS-1.

This study has several limitations*.* Owing to the *post hoc* nature of the analysis, not all data were available from all three studies (*e.g.*, the time of RRT in the REVERSE study^12^), which precluded RRT-free survival analyses and competing risk and hierarchical analyses in the pooled population. In addition, the duration of follow-up did not extend beyond 90 days, which prevented evaluation of longer-term outcomes. Finally, the studies were designed before the adoption of the current HRS-AKI diagnostic criteria proposed by the International Club of Ascites in 2015, which defined HRS-AKI as a doubling in sCr in 14 days, rather than a fixed threshold of sCr, allowing for an earlier detection and treatment of HRS-1.^15^ Consequently, we could not evaluate the effect of terlipressin on RRT when implemented as an early treatment for HRS-1.

Despite the limitations, our findings expand on the knowledge regarding the therapeutic benefit of terlipressin in patients with HRS-1 presented in the primary analyses of the OT-0401, REVERSE, and CONFIRM studies.^11–13^ Extensive evidence in the literature demonstrates that AKI-RRT is associated with more ominous acute and long-term outcomes compared with AKI without the need for RRT.^5,20,27^ Therefore, any medication that reduces the need for RRT should be considered favorable for patients with cirrhosis and HRS-1.

The results from this *post hoc* analysis affirm that pharmacological intervention with terlipressin plus albumin may reduce the need for RRT in patients with HRS-1, including among those who receive an LT. Association of a lower sCr value at the beginning of therapy with avoidance of RRT may support an earlier start for HRS-1 treatment initiation (*i.e.*, before sCr rises to a level requiring RRT).

## Data Availability

Discussion of statistical end points and analysis are included in the article. Summary aggregate (basic) results (including adverse events information) and the study protocol will be available on ClinicalTrials.gov (CONFIRM, NCT02770716) when required by regulation. Individual deidentified patient data will not be disclosed. Requests for additional information should be directed to the company at medinfo@mnk.com.

## Supplemental Material

Supplemental Table 1. Baseline patient demographics and clinical characteristics for the CONFIRM, OT-0401, and REVERSE studies; ITT populations.
